# Practical Laboratory Points

**Published:** 1896-08

**Authors:** J. G. Templeton

**Affiliations:** Pittsburg, Pa.


ARTICLE III.

### PRACTICAL LABORATORY POINTS.

BY J. G. TEMPLETON, D. D. S., PITTSBURG, PA.

I am inclined to think that any testimony or description the writer may give of practical laboratory points will appear so blunted that they can be sharpened by any ordinary dentist.

In the dental laboratory it is very important to give particular attention to little things, which, of themselves, seem to be minor. Yet, if omitted, these little things often show much defect in the final results. If called on to give advice to all dentists, and particularly to the younger men in the profession, it would be to get all the little practical points possible, and store them on a shelf in memory that they may be ready for use when needed, like a good friend in time of perplexity and trouble.

A slip noose can be put on the lower front teeth with one hand while the rubber-dam is held down with the other. Get your slip knots ready first, draw them tight, and they will hold on as long as wanted.

To solder a cap on a gold tube attached for an artificial crown, lay the cap on about a tablespoonful of finely-cut asbestos, put the tube in place on the cap, drop in the solder and a little powdered borax, then blow a yellow flame on the asbestos all around the tube till the solder flows. There will be no danger of melting the gold.

In vulcanite work the best results may be obtained by making models one-fourth marble dust and three-fourths plaster; also the same in flasking the case.

To keep rubber dam from running between the teeth and joints in vulcanizing, after the teeth are set in the first half of the flask plaster, trimmed and varnished, pour water on all the teeth and joints, then mix a small quantity of pure plaster, have it rather thin, and with mixing spatula cover labial and buccal surfaces, also the joints; take up the piece quickly and bring it near the mouth and blow rather sharply against the thin plaster all around, which will force it into all spaces between the teeth or blocks. After this finish, flasking in the usual way, and, if possible, it is well to allow the case to remain over night in the flask before packing.

To keep plaster from sticking to palatine surface of plate just before beginning to pack the case, coat the model with a thick lather of good soap. In finishing the plate, always trim the rim low over the bicuspids, leaving it high as can be worn over the cuspids, and the same over and back of the second molars; do not file rim to a knife-like edge, slightly bevel inside of rim at the top extending down about three-sixteenths of an inch.

To make platina and gold plate, melt with blow-pipe pure gold on a piece of platina and roll to the desired thickness, the result will be as good as any you can buy, and you will have saved at least thirty cents per pennyweight.

United States gold coin is 21 6-10 k. fine. Instead of buying 22k. plate from the supply houses for crown- and bridge-work, get United States gold coin (the older coins not alloyed with copper are best), and you will save $1.50 on each $5 worth. A $5 gold piece weighs five pennyweights and ten grains.

Much can be saved by the dentist making his own solders. Good formulas are to be found in both Harris and Richardson; also elsewhere. We have for several years used a formula obtained from Dr. Melotte, of Ithaca, N. Y., which is as follows :

Take a United States $5 gold piece, 20 grains coin silver, 10 grains pure copper, 6 grains English toilet pins; melt the silver and copper together first, after melting this and the gold together, add the pins, flow into an ingot and roll, cut it into small pieces and melt again if it should not roll well first time, this will give a solder a little more than 19k. fine, and flows nicely on coin gold, being the same color.

This we call No. 1. Now take of No. 1:

| | | |
|---|---|---|
| No. 1 | . . . | 89 grs. |
| Coin silver, | . . | 7 grs. |
| Pure copper, | . . | 4 grs. |

Melt together and roll, and we have a second grade which we call No. 2, and which will flow on No. 1.

To make a still lower grade, take:

| | | |
|---|---|---|
| Pure gold, | . . | 6 dwt. |
| Copper, | . . . | 2 dwt. |
| Fine silver, | . . | 1 dwt. |

And you will have a 16k. solder. In my practice only Nos. 1 and 2 are used,—*Dental Review.*

